# The outcome of skeletofacial reconstruction with mandibular rotation for management of asymmetric skeletal class III deformity: A three-dimensional computer-assisted investigation

**DOI:** 10.1038/s41598-019-49946-9

**Published:** 2019-09-16

**Authors:** Ting-Yu Wu, Rafael Denadai, Hsiu-Hsia Lin, Cheng-Ting Ho, Lun-Jou Lo

**Affiliations:** 1 Division of Orthodontics, Department of Dentistry, Chang Gung Memorial Hospital, Taoyuan, Taiwan; 2 Department of Plastic and Reconstructive Surgery and Craniofacial Research Center, Chang Gung Memorial Hospital, Chang Gung University, Taoyuan, Taiwan; 3 Image Lab and Craniofacial Research Center, Chang Gung Memorial Hospital, Chang Gung University, Taoyuan, Taiwan

**Keywords:** Bone imaging, Outcomes research

## Abstract

The mandibular proximal ramus segments should be moved and rotated during orthognathic surgery-based skeletofacial reconstruction for the correction of challenging patients with facial asymmetry and malocclusion, but quantitative data regarding this rotation were not sufficient to date. This 3D computer-assisted study measured the proximal ramus segment rotation after 3D simulation-guided two-jaw surgery in patients with facial asymmetric deformity and class III malocclusion (n = 31). Using 3D mandible models and a reliable proximal ramus segment-related plane, angular changes in pitch, roll and yaw directions were measured before and one month after surgery. Significant rotational changes (p < 0.01) were observed in the left and right sides and overall proximal ramus segments after surgery, with absolute differences of 4.1 ± 3.0 (range −7.8 to 6.9), 2.8 ± 2.3 (−8.8 to 5.0), and 2.7 ± 2.4 (−6.6 to 9.9) degrees in pitch, roll, and yaw rotations, respectively. Numbness and mouth opening limiting occurred within the first 6 months after surgery but the patients had an unremarkable long-term postoperative course, with no revisionary surgery required. This study contributes to the multidisciplinary-related literature by revealing that proximal ramus segment rotation and rigid fixation with no postoperative intermaxillary immobilization was practicable in skeletofacial surgery for the successful treatment of asymmetric deformity and class III malocclusion.

## Introduction

Skeletofacial surgery is an effective procedure for patients with challenging cases of facial asymmetry and malocclusion^1–4^. Positions of the maxilla and mandible, especially the ramus and chin, are frequently found to be deviated in individuals with facial asymmetry^4^. Traditionally, the intraoperative manipulation of the proximal ramus segment has mainly been based on the surgeon’s experience^5,6^. As preoperative misdiagnosis and inaccurate planning may contribute to unsuccessful results with the need for redo orthognathic surgery-based skeletofacial reconstruction or revisionary procedures, three-dimensional (3D) simulation has been recognized as a key factor to achieve consistent success in skeletofacial surgical treatment^7–12^. In the 3D planning of facial asymmetry correction, the final maxillomandibular complex incorporates the horizontal, anteroposterior, and vertical translations as well as pitch, roll and yaw rotations^13^. Using the bilateral sagittal split osteotomy (BSSO) technique, the proximal ramus segment should also be rotated in the three potential directions to achieve a symmetric and balanced face, with center of rotation at the condyle. Alternatively, others have reported the use of the intraoral vertical ramus osteotomy (IVRO) technique but requiring postoperative intermaxillary immobilization^14,15^.

In this setting, the postoperative changes of the proximal ramus segment have been only sparsely investigated in patients with no evident facial asymmetry by using two-dimensional image-based measurement methods. Limited reports of the condylar head movement in a linear direction (≤1 mm) with some extent of rotation have previously been described^14,16–21^. It was also shown that the condylar angle changes in all three rotational planes after skeletofacial surgery^17,22–26^, indirectly indicating that the position of the proximal ramus segment was altered, and rotation had occurred. Moreover, this postoperative rotation could produce temporomandibular (TMJ) disorders^27,28^.

The establishment of 3D technology-based quantitative data related to the rotation of the proximal ramus segment after surgery may provide helpful information for multidisciplinary teams (dentists, orthodontists, oral surgeons, maxillofacial surgeons, ear, nose and throat surgeons, head and neck surgeons, and plastic surgeons) in surgical planning and execution. The purpose of this 3D computer-assisted study is to measure the postoperative rotation of the proximal ramus segment in a sample of patients with facial deformity managed with two-jaw surgery, using the BSSO mandible setback technique with bicortical screws-based rigid fixation and no postoperative intermaxillary immobilization.

## Results

### Proximal segment rotation measurement

On average, the right and left mandible sides and overall proximal ramus segments had a postoperative rotation of 4.1° each in pitch and 3.3°, 2.4°, and 2.8° in roll, and 3.0°, 2.3°, and 2.7° in yaw directions, respectively. On T0 versus T1 comparative analyses, significant differences (all p < 0.05) were observed for all three types of proximal segment rotations in the right and left sides and overall proximal ramus segments (Table 1). The proximal segment plane (PSP) presented more angle reduction in pitch and roll directions, and more angle increase in yaw direction (Table 1). The intra-examiner reliability was excellent (ICC = 0.904–0.982) for all landmark localizations and measurements (Table 2).

**Table 1:** Rotation of the Proximal Ramus Segment after Skeletofacial Surgery.

Parameters	Rotation of the Proximal Ramus Segment		
	Pitch *	Roll^†^	Yaw^‡^
**Right side** (***n*** = **31**)			
T0, m ± sd	72.1 ± 4.6	80.5 ± 4.8	12.8 ± 5.4
T1, m ± sd	70.6 ± 6.7	78.7 ± 5.6	14.2 ± 5.7
Absolute difference overall, m ± sd (r)	4.1 ± 3.1 (−7.8–6.9)	3.3 ± 2.5 (−8.6–5.0)	3.0 ± 2.4 (−6.6–8.2)
Absolute difference for negative values, m ± sd (r)	4.5 ± 3.4 (−7.8–−0.4)	4.4 ± 2.5 (−8.6–−0.6)	2.2 ± 2.1 (−6.6–−0.4)
Absolute difference for positive values, m ± sd (r)	3.3 ± 2.3 (0.8–6.9)	1.7 ± 1.6 (0.3–5.0)	3.5 ± 2.4 (0.3–8.2)
*p*	0.048	0.005	0.014
**Left side** (***n*** = **31**)			
T0, m ± sd	72.1 ± 5.2	83.0 ± 4.2	9.1 ± 3.7
T1, m ± sd	70.4 ± 7.0	81.6 ± 4.5	10.3 ± 4.8
Absolute difference, m ± sd (range)	4.1 ± 2.6 (−7.6–6.5)	2.4 ± 1.9 (−8.8–4.6)	2.3 ± 2.3 (−4.0–9.9)
Absolute difference for negative values, m ± sd (r)	4.3 ± 3.3 (−7.6–−0.2)	2.6 ± 2.1 (−8.8–−0.3)	1.5 ± 1.6 (−4.0–−0.3)
Absolute difference for positive values, m ± sd (r)	3.7 ± 2.1 (1.1–6.5)	1.8 ± 1.3 (0.9–4.6)	2.7 ± 2.5 (0.4–9.9)
*p*	0.028	0.006	0.015
**Overall proximal segments** (***n*** = **62**)			
T0, m ± sd	72.1 ± 4.9	81.7 ± 4.6	10.9 ± 4.9
T1, m ± sd	70.5 ± 6.8	80.1 ± 5.3	12.3 ± 5.6
Absolute difference, m ± sd (range)	4.1 ± 3.0 (−7.8–6.9)	2.8 ± 2.3 (−8.8–5.0)	2.7 ± 2.4 (−6.6–9.9)
Absolute difference for negative values, m ± sd (r)	4.4 ± 3.3 (−7.8–−0.2)	3.4 ± 2.4 (−8.8–−0.3)	1.8 ± 1.8 (−6.6–−0.3)
Absolute difference for positive values, m ± sd (r)	3.5 ± 2.2 (0.8–6.9)	1.7 ± 1.4 (0.3–5.0)	3.1 ± 2.5 (0.3–9.9)
*p*	0.00324	0.00012	0.00009

m, mean; sd, standard deviation; r, range; T0, before surgery; T1, one month after surgery; absolute difference, the mean absolute difference between T0 and T1.

*Negative values indicate counterclockwise rotation, whereas positive values clockwise rotation viewing from the axis.

^†^Negative values indicate medial rotation, whereas positive values lateral rotation viewing from the axis.

^‡^Negative values indicate outward rotation, whereas positive values inward rotation viewing from the axis.

**Table 2:** Intra-Examiner Reliability Values.

Landmarks	Axes	ICC	
		Right side	Left side
PR1-T0	X-axis	0.954	0.943
	Y-axis	0.968	0.955
	Z-axis	0.982	0.946
PR1-T1	X-axis	0.904	0.922
	Y-axis	0.930	0.943
	Z-axis	0.925	0.939
PR2-T0	X-axis	0.947	0.921
	Y-axis	0.965	0.934
	Z-axis	0.956	0.944
PR2-T1	X-axis	0.944	0.954
	Y-axis	0.962	0.932
	Z-axis	0.946	0.954
DSN-T0	X-axis	0.912	0.946
	Y-axis	0.957	0.929
	Z-axis	0.946	0.915
DSN-T1	X-axis	0.947	0.943
	Y-axis	0.943	0.962
	Z-axis	0.939	0.958

T0, before surgery; T1, after surgery; ICC, intraclass correlation coefficients. For PR1, PR2, and DSN definitions, please refer to Table 5.

### Skeletofacial surgery-related complaints and complications

**Figure 1:**
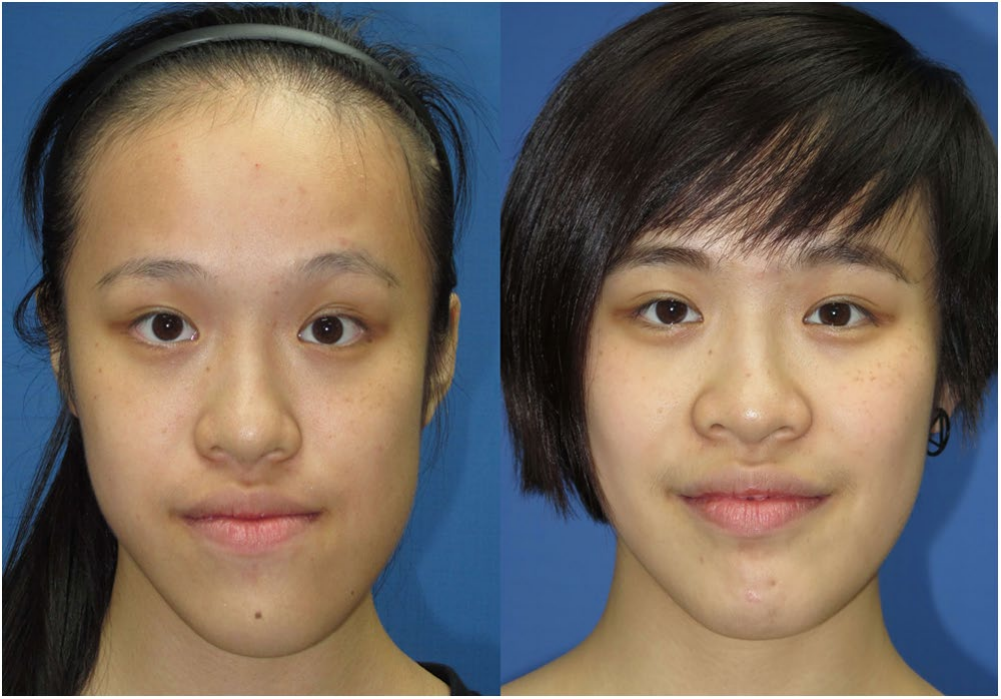
(Left) This 18-years old female patient with prognathism, facial asymmetry, and long face (right) received Le Fort I, bilateral sagittal split of ramus, and genioplasty.

**Figure 2:**
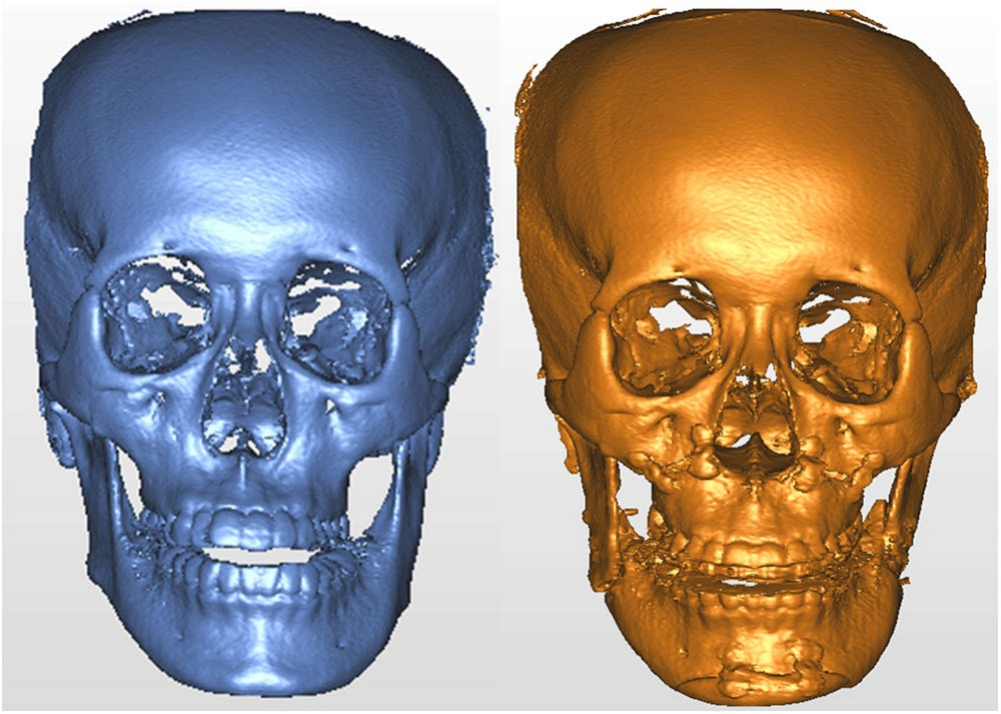
(Left) Preoperative and (right) postoperative cone-beam computed tomography-based craniofacial images of same patient as in Fig. 1. Postoperative measurement of the rotation showed 4.5° and 3.8° in pitch, 3.6° and 7.4° in roll, and 4.4° and 4.9° in yaw on her right and left planes of proximal segment (PSPs), respectively.

Fifty-five percent of patients presented with chin or lip numbness at 1-month postsurgery, with full recovery at long-term evaluations. Two patients had hypersensitivity over the chin area 1 year after surgery. One patient had bloody discharge and wound infection which was successfully controlled by antibiotics (Table 3). For TMJ-related conditions, all patients were asymptomatic before surgery. Eighty-seven percent of patients had mouth opening limitation (<4 cm) at 1-month postsurgery, with satisfactory interincisal mouth opening achieved between 6 and 12 months after surgery. One patient reported TMJ soreness during the first 6 months postsurgery (Table 3) but the clinical and radiographic examinations showed no positive findings. All patients were satisfied with the results, with no request or indication for revisionary surgery in this cohort (Figs 1 and 2).

**Table 3:** Post-Skeletofacial Surgery Related Signs and Symptoms (*n* = 31).

Parameters	Post-Skeletofacial Surgery			
	1 month	3 months	6 months	≥12 months
**Complications** *n* (%)				
Infection	1 (3)	—	—	—
Bleeding	1 (3)	—	—	—
Hypersensitivity	—	—	—	2 (6)
Numbness	17 (55)	12 (39)	7 (23)	1 (3)
**TMJ features** *n* (%)				
Clicking sound	—	—	—	—
Pain or tenderness*	1 (3)	1 (3)	1 (3)	—
Mouth opening < 4 cm	27 (87)	10 (33)	3 (9)	—
Headache	1 (3)	—	—	—
Tinnitus	—	—	—	—

n, number of patients; TMJ, temporomandibular joint; *, in the TMJ area.

## Discussion

The orthodontic-surgical technical details (virtual planning, modified surgery-first approach, single splint technique, two-jaw surgery and genioplasty, and with no postoperative intermaxillary immobilization) adopted in this study have been beneficial not only for correction of the occlusal function, but also to achieve facial symmetry, balance, proportion, and improved aesthetics in successive cohorts of skeletofacial surgery-treated patients at our center in recent years^11,12,29–40^. For the particular planning and execution of facial asymmetry correction, 3D simulation helps to demonstrate the rotation of both proximal and distal ramus segments with maintenance of intersegmental space and angulation between proximal and distal segments to achieve the desired facial symmetry. The postoperative amount of this rotation had not been sufficiently investigated to date.

In the current investigation, the extent of rotation of the proximal ramus segment in pitch, yaw, and roll directions was measured by employing a 3D computer-based proximal ramus-specific plane process with a mixed landmark and best-fist method in patients who had visible facial asymmetry with occlusal cant and chin deviation. For this, the difference of the PSP-related points between pre- and post-surgery mandible models was based on integrated 3D models of T0 and T1 (pre- and post-surgery, respectively) by using the surface best-fit method (superimposed on forehead and orbital areas). Before the proximal ramus rotation-related data collection, the precision of this superimposition was ascertained in the upper skeletofacial framework which had no influence of surgery by the color map and root-mean-square deviation (RMSD) tools. Color map- and RMSD-related values and reliability tests revealed that the virtual-guided data collection was accurately and consistently performed. This study contributes to literature by demonstrating that the rotation of the proximal ramus segment in a range within 10° for each of the three directions has resulted not only in the achievement of the desired facial symmetry with no need for revisionary surgery, but also in successful functional results with no compromise of the occlusion and TMJ function, as revealed in long term follow-up evaluations after debonding. This was attained by using a BSSO mandible setback approach along with a three bicortical screws-based rigid fixation technique, with no need of intermaxillary immobilization postsurgery, attenuating the overall mouth open restriction-related stress for patients and their parents during the postoperative period as well as improving wound care with intensive oral hygiene started immediately after anesthetic recovery.

In the relevant literature, direct or indirect rotational changes of the ramus after skeletofacial surgery in patients with skeletal class III and facial asymmetry are also reported^5,6,13,14^, but differences in sample composition (inclusion of patients with no facial asymmetry), surgical techniques (BSSO and IVRO), and measuring tools (cephalometric- or condyle-based methods) impair a truthful head-to-head comparison between the existing findings and our current results^5,6,13,14^. Cephalometric- and condyle-based quantitative data might indirectly show the rotation of the proximal ramus segment but it does not provide accurate information to help the decision-making process in the planning and execution of ramus mobilization in the three potential directions as a need-based maneuver to improve facial contour symmetry. The current 3D-based proximal segment-specific numerical findings may therefore act as a reference for preoperative planning and intraoperative guiding of rotational changes of the proximal ramus segment to achieve a balanced occlusion and TMJ functional and facial symmetric result, with no long-term TMJ-related signs and symptoms.

When planning and treating facial asymmetry, yaw rotation of the maxillomandibular complex was frequently performed in order to reach facial symmetry and avoid bony collision^9,10,39,40^. The roll rotation (medial-lateral movement) of the proximal ramus segment was performed accordingly, but some limitation on yaw rotation in the proximal segment was attempted during the bicortical screw fixation. For such yaw rotation, we showed an average 2.7° absolute difference, with the overall proximal ramus segments showing a mean increase of 1.4° after surgery (10.9 for T0 vs. 12.3 for T1). Evaluating the roll rotation after BSSO for treatment of facial asymmetry, a previous study found a mean change of 1.5° in medial movement^19^. From our findings, the average change was of 1.6° (81.7 vs. 80.1) in medial rotation for overall proximal ramus segments, with values ranging from 5.0° to −8.8° for lateral and medial rotations, respectively. Particularly for managing patients with prominent facial deviation, roll rotation of the proximal ramus was a key step in order to achieve cheek and contour symmetry (Video S1). In this situation, it was common that one side of ramus was moved inward and the other side moved outward.

It is controversial whether the pitch clockwise rotation of the proximal ramus segment could increase the relapse in BSSO-based mandibular setback, with positive^41,42^ and negative^21,43^ correlations being reported. Appraising pitch rotation of the proximal segment using a condyle-based sagittal view method, another group reported a significantly decreased angle (counterclockwise rotation) with a mean of 2.32° after BSSO mandibular setback^2^. We demonstrated similar findings for mean changes with right and left mandible sides presenting 1.5° (72.1 versus 70.6) and 1.7° (72.1 versus 70.4) in counterclockwise pitch rotation, respectively. Additionally, our data also show that the pitch-related values ranged from 7.8° in counterclockwise to 6.9° in clockwise rotations for overall proximal segments, with clinically acceptable occlusal status in the 1-year follow-up after debonding. Perhaps multiple factors are involved in mandibular stability after BSSO setback, rather than just the pitch rotation in the proximal ramus segment^44,45^.

In contrast to previous studies, we used absolute difference values to show the rotation on the proximal ramus segment after skeletofacial surgery, with the average of 4.1°, 2.8°, and 2.7° in pitch, roll, and yaw rotations for overall proximal segments, respectively (Table 1). The range of rotation angle indicated the tolerance of movement without creating clinical problems in occlusion, TMJ, and facial contour parameters. From previous BSSO-based recommendations for surgical simulation, the proximal ramus segment rotation should be manipulated within 4° and 3° in pitch and roll directions, respectively^6^. Our overall findings support this prior reference guide, but for patients with severe facial asymmetry, the clinically acceptable tolerance to additional rotational changes was also observed. These ranging, values-related findings may be specifically valuable for multidisciplinary teams managing difficult clinical scenarios such as malocclusion associated with severe facial asymmetry.

The most frequent postoperative complaints and complications in our patients were numbness and limitation in mouth opening, which resolved in 6 to 12 months postsurgery (Table 3). These are to be expected after skeletofacial surgery, but these clinical repercussions should always be attenuated by surgical maneuvers and postoperative care^37,46,47^. Other TMJ-related concerns were uncommon in our cohort, supporting a former report^21^. While the patient-centered outcome of the presence of TMJ-related symptoms and signs is a useful and relevant outcome measure^21,37,46,47^, further studies may expand the current findings by inclusion of objective evaluations.

Limitations of this study include inherent bias associated with a non-comparative retrospective design. We included different degrees of facial asymmetry, but the full spectrum of potential conditions was not addressed. It was restricted to nonsyndromic young adult patients with class III malocclusion, as it reflects our skeletofacial surgery population^11,12,30–34^. The potential translational displacement of condylar head was considered minimal and disregarded in our study. Reports have described a condyle move of 0.36 mm inferiorly and 0.03–0.58 mm anteriorly after BSSO mandible setback^16,19^. In our study, the condyle was consistently returned into the glenoid fossa before rigid fixation. A trivial condylar displacement could happen, but the movement was not determinant in causing clinical repercussions such as postoperative TMJ-related symptoms and malfunctioning, malocclusion, or residual facial asymmetry. Further investigation may address this issue by applying our 3D cone beam computed tomography (CBCT) image-based method for proximal ramus segment plane-specific measurements alongside the condyle-based measurement method. The exact cut off point for rotation of the proximal ramus segment according to each particular type of facial asymmetry may also be further investigated to create a patient-specific skeletofacial surgery approach.

In conclusion, this study provides 3D-based measurements of rotational changes of the proximal ramus segment in patients with facial asymmetry and class III malocclusion, with acceptable TMJ and occlusion functional and facial symmetric results.

## Patients and Methods

### Study population

This Institutional Review Board-approved (Chang Gung Medical Foundation, protocol 103–2822 B) retrospective study recruited consecutive patients with facial deformity (developmental facial asymmetry and class III malocclusion) who underwent orthodontic treatment and orthognathic surgery by the two senior authors (C.-T.H. and L.-J.L.) at the Chang Gung Craniofacial Research Center between July 2014 and July 2017. All experiments and the study methods were carried out in accordance with the approved guidelines of Institutional Review Board. Written informed consents were obtained from the patients or the guardians of the patients younger than 20 years and image release forms for clinical pictures were obtained accordingly for all patients displayed in this publication.

Demographic, clinical, surgical, and outcome data were collected. Patients were excluded if they had cleft or associated syndromes, had a history of facial surgery, did not undergo adequate 3D imaging, or did not complete the follow-up observation (<12 months after debonding).

The included sample comprised 17 women and 14 men with a mean age of 21.4 years. In clinical evaluation, all 31 patients had a concave facial profile, paranasal depression, and protruding mandible with deviation. Radiographic images revealed a class III skeletal relationship, a negative ANB angle, and negative overjet (Table 4). Facial asymmetry was visible and characterized by occlusal plane canting, a discrepancy between the upper and lower dental midlines, cheek asymmetry, and chin deviation with menton at least 4 mm away from the facial midline^48^.

**Table 4:** Preoperative Cephalometric Measurement (T0) in the Included Patients (*n* = 31).

Parameters	Values (m ± sd)
**Lateral view**	
SNA (degrees)	81.62 ± 3.82
SNB (degrees)	84.79 ± 4.12
ANB (degrees)	−3.16 ± 2.45
Overjet (mm)	−4.68 ± 4.01
**Frontal view**	
Occlusal canting (degrees)	2.16 ± 2.11
Ramus inclination difference (degrees)	4.71 ± 2.76
U1-MSL (mm)	1.52 ± 1.13
L1-MSL (mm)	3.35 ± 1.80
Me-MSL (mm)	5.32 ± 2.49

mm, millimeters; m, mean; sd, standard deviation; SNA, angle between SN and NA line; SNB, angle between SN and NB line; ANB, angle between AN and NB line; overjet, the horizontal distance between incisal edges of upper and lower central incisors; ramus inclination difference, the absolute difference of angle between right and left ramus inclination in frontal view; U1, incisal edge of upper central incisor; L1, incisal edge of lower central incisor; MSL, mid-sagittal line; Me, menton.

### Surgical treatment approach

All the included patients received single-splint two-jaw surgery with or without genioplasty according to the previously described CBCT-guided virtual surgical planning (Dolphin 3D software, Dolphin Imaging & Management Solutions, California, USA) and surgical approach principles^11,12,30–34^. In this center, standard CBCT scans have been performed 2 weeks preoperatively for accurate diagnostic evaluation and surgical planning and 1 month postoperatively for assessment of surgical skeletal changes, which act as guidance for postoperative patient-specific orthodontic-surgical care. Presurgical orthodontic treatment was performed for arch form compatibility and took 6.5 ± 3.2 months, including leveling, alignment, arch coordination, and dental decompensation. The orthodontic treatment continued after surgery.

**Figure 3:**
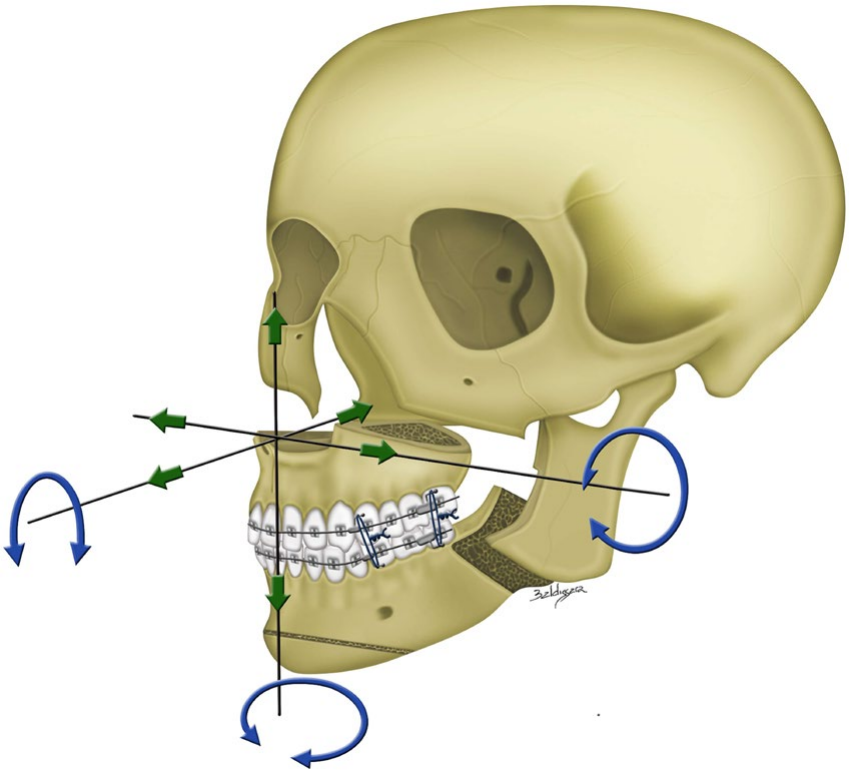
Single-splint two-jaw orthognathic surgery-based skeletofacial reconstruction principle. (a) Both maxilla (Le Fort I segment) and mandible (two proximal ramus segments and one distal segment) were completely osteotomized, fixed in the final occlusion (surgical occlusion splint), and moved as an integrated “maxillo-mandibular complex” (MMC) to the 3D-simulated position. To transfer the virtual simulation to actual surgery, the MMC was moved in six potential directions, including pitch, roll, and yaw rotations (blue arrows) and en-bloc linear horizontal (left or right shifts and advancements or setbacks in the antero-posterior direction) and vertical (extrusion or intrusion) movements (green arrows).

**Figure 4:**
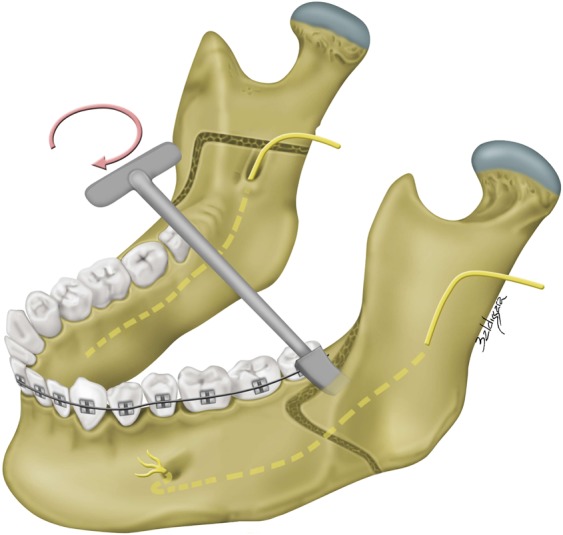
A modified Obwegeser-Dal Pont bilateral sagittal split osteotomy (BSSO) technique was adopted in all included patients. After lingual and buccal corticotomies (3-mm spherical bur and Lindeman side-cutting bur) and sagittal osteotomy (reciprocating saw), a Dautrey osteotome was passed beyond the location of inferior alveolar nerve under direct visualization through anterior opening and driven to complete the splitting of the mandibular ramus.

To transfer the 3D planning to actual surgery (Figs 3 and 4), measurements in maxillary pillars bilaterally, face bow-based midline checking (nasal dorsum and tip, lips, maxilla, dental arches, and chin areas), and middle and lower facial third proportions judgments were used as reference. The splitting of BSSO was carefully accomplished to avoid injury to the inferior alveolar nerve (Fig. 3). The maxillomandibular complex with final surgical splint was moved to the desired position (Fig. 4). After Le Fort I fixation with miniplates and screws, the proximal ramus segment was placed in a relaxed position and gently pushed up to ensure the position of the condylar head in the glenoid fossa. Using the 3D simulated image as a guiding template, the desired relationship between the proximal and distal segments was achieved. Percutaneous insertion of 3 bicortical screws 14–16 mm long was performed in the ramus. No interpositional bone graft was used in procedures requiring maintenance of intersegmental gaps. Intermaxillary fixation was released and the occlusion was evaluated. Genioplasty was finally executed as planned, along with intraoperative judgement.

### 3D image acquisition

Preoperative (2 weeks before surgery, T0) and postoperative (1 month after surgery, T1) 3D maxillofacial images were acquired using an i-CAT CBCT scanner (Imaging Sciences International, Hatfield, PA, USA) with a low-dose protocol and patient teeth under a light contact condition (120 kV, 5 mA, and 50 Hz). The extended field of view was 22 (height) × 16 (depth) cm, scanning time was 40 s, and voxel size was 0.4 × 0.4 × 0.4 mm. 3D mandible study models were processed and analyzed using the SimPlant O&O software program (Materialize, Leuven, Belgium). By using the standard segmentation function, the skull and the bilateral proximal segments of ramus were outlined.

### Anatomical landmarks identification and selection

A threshold segmentation was used for CBCT-based 3D model reconstruction obtained from DICOM (Digital Imaging and Communications in Medicine) files by identifying and delineating the anatomic structures of interest in the CBCT-derived image. The threshold was chosen by an experienced health bioinformatics specialist, and the same skeletofacial landmarks were identified twice, namely on the 3D model and in each slice (for a practical example refer to Supplementary Fig. S1). The distance between the same landmarks on the 3D model and in each slice was then calculated to check the right threshold value that secures the landmarks identification later on (Supplementary Fig. S2). For the tip of coronoid process (TCR) point identification and selection, we adopted the surface of CBCT-based 3D model with an interactive checking of the grayscale in each sliced image (Supplementary Fig. S3). It was initially performed in T0 models and then transferred to T1 models by the best-fit method for identifying the exact same TCR point.

### Defining the plane for proximal segment

**Figure 5:**
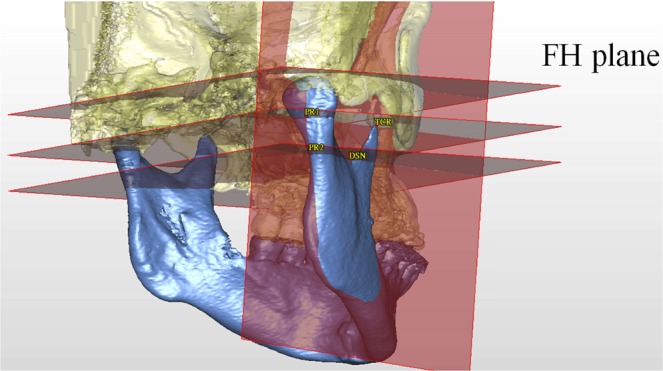
Designing the representing plane for proximal ramus segment. Landmarks showed the tip of coronoid process (TCR), deepest point of sigmoid notch (DSN), PR1, and PR2 (definitions in Table 5). The plane of proximal segment (PSP) was defined by PR1, PR2, and DSN points. FH plane means Frankfurt horizontal plane.

In order to consistently measure the rotation of the proximal ramus segment in both T0 and T1 mandible models, a representing plane should be created. For this, Frankfurt horizontal plane (FHP)^35^, TCR point, and the deepest point in the sigmoid notch (DSN) were demarcated (Table 5). Using these reference plane and points, two further landmark points (PR1 and PR2) which were not influenced by the BSSO in ramus were defined on the posterior side of the proximal ramus segment (Fig. 5). A consistent reference plane representing the proximal segment (PSP) of ramus was then defined by PR1, PR2, and DSN points (Fig. 5, Table 5).

**Table 5:** Definition of the Landmarks, Reference Planes, and Measurement of Rotation.

Items	Definitions
Frankfurt horizontal plane (FHP)	The plane passes the bilateral porion points and the midpoint between the bilateral inferior orbital points^35^
Midsagittal plane (MSP)	The plane perpendicular to the FHP and passing through the sella turcica (S) and the nasion (N) points
TCR	The point at the tip of coronoid process
DSN	The deepest point in the sigmoid notch
PR1	The point at the most posterior side of the ramus at the junction of a plane passing the TCR point and paralleling the FHP
PR2	The point at the most posterior side of the ramus at the junction of a plane passing the DSN point and paralleling the FHP
PR1-PR2	A plane passing through PR1, PR2 and perpendicular to MSP
Proximal segment plane (PSP)	Defined by PR1, PR2, and DSN points
Pitch rotation	The angle of rotation around mesial-distal axis on skull measured by changes of PR1-PR2 and FHP
Roll rotation	The angle of rotation around anterior-posterior axis on skull measured by changes of PSP and FHP
Yaw rotation	The angle of rotation around vertical axis on skull measured by changes of PSP and MSP

### Proximal segment rotation measurement

**Figure 6:**
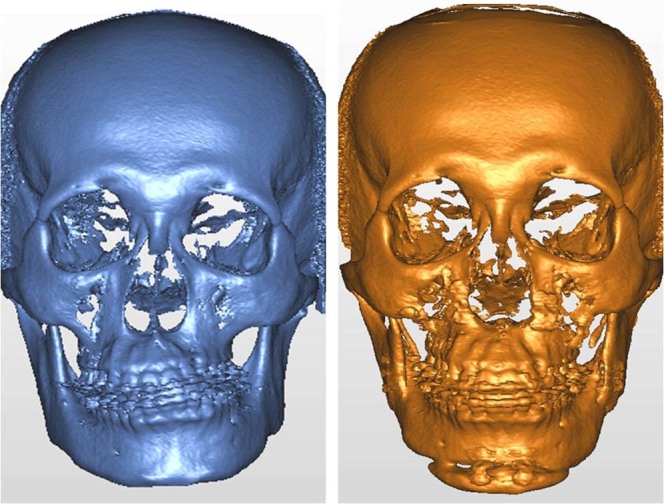
A clinical example of facial asymmetry and chin deviation (left) before and (right) after skeletofacial surgery.

**Figure 7:**
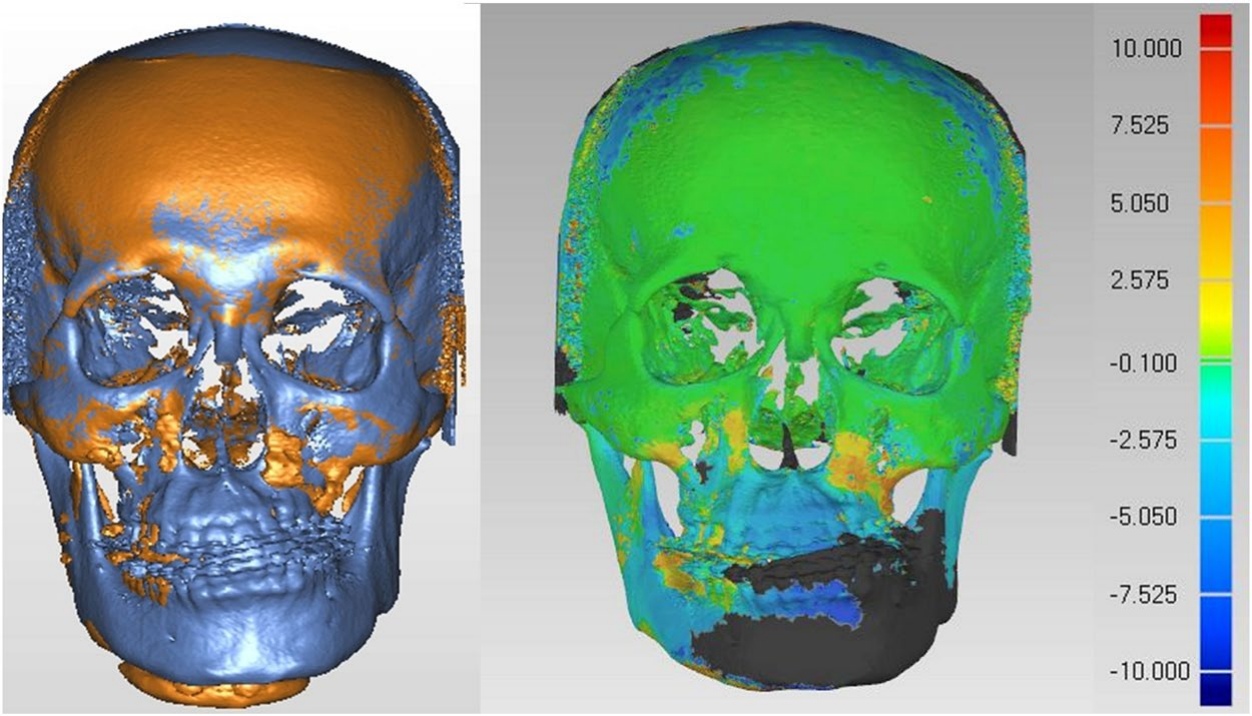
T0 preoperative and T1 postoperative images of same clinical example as in Fig. 4. (Left) T0 and T1 images were superimposed on frontal and supraorbital bone. (Right) The color map indicated accurate registration of the two models on frontal and supraorbital bone showing the green color. The magnitude of the differences was greater in the lower facial parts, demonstrating the operative change.

**Figure 8:**
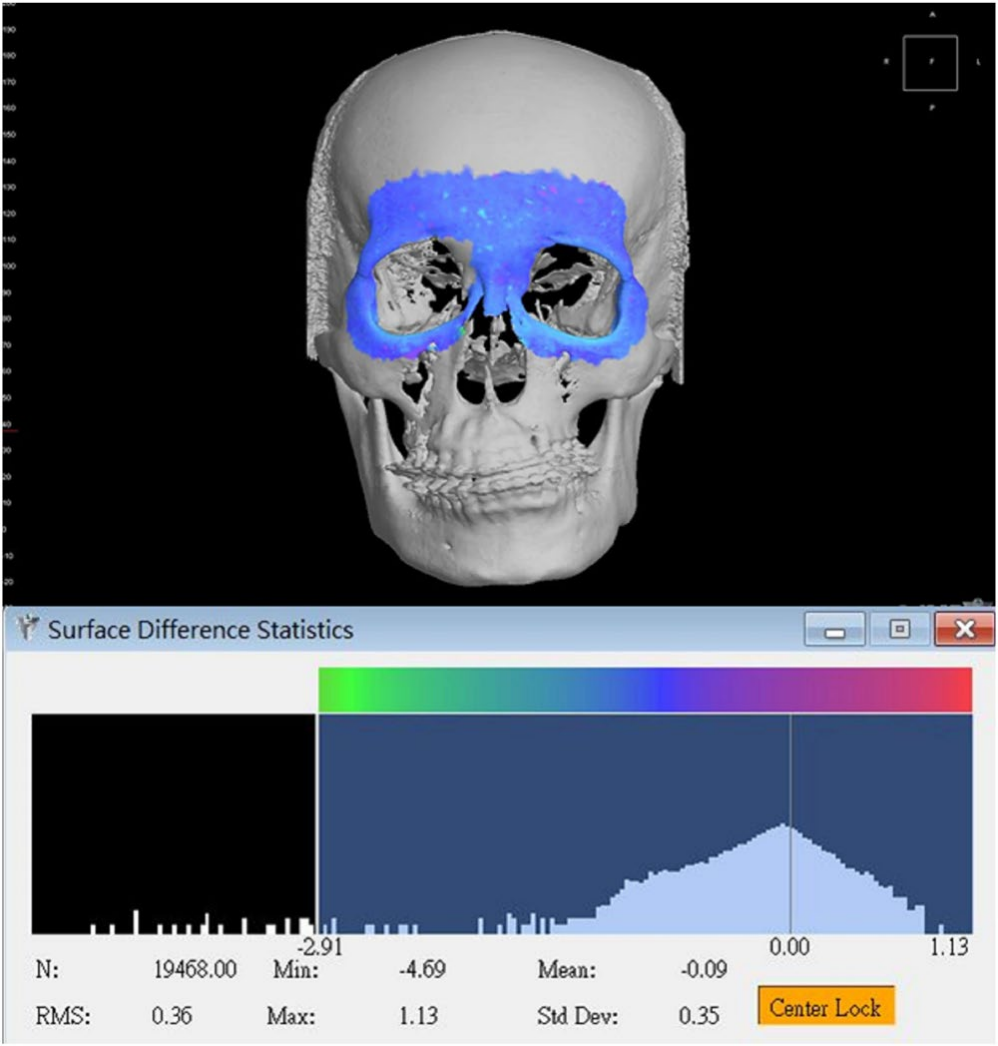
Root-mean-square deviation (RMSD) was used to measure the accuracy of image superimposition, with value inferior to 0.5 mm considered acceptable. Same superimposed model as in Fig. 7.

**Figure 9:**
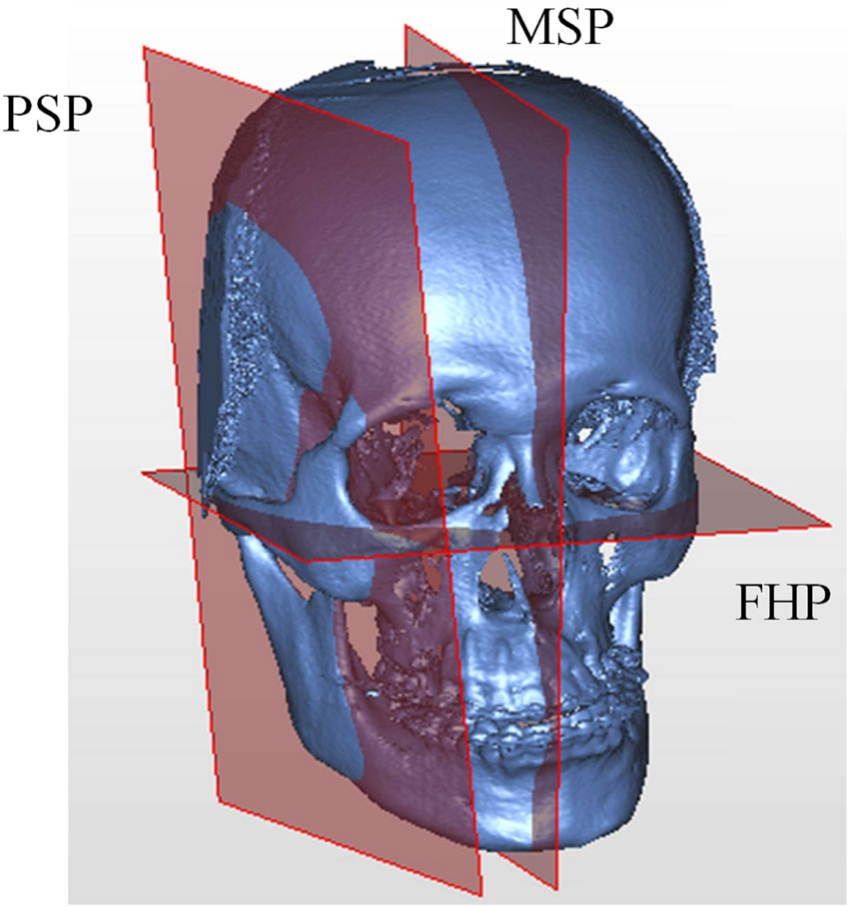
The coordinated reference system. Frankfurt horizontal plane (FHP) and midsagittal plane (MSP) were constructed, and the plane of proximal segment (PSP) was formed.

**Figure 10:**
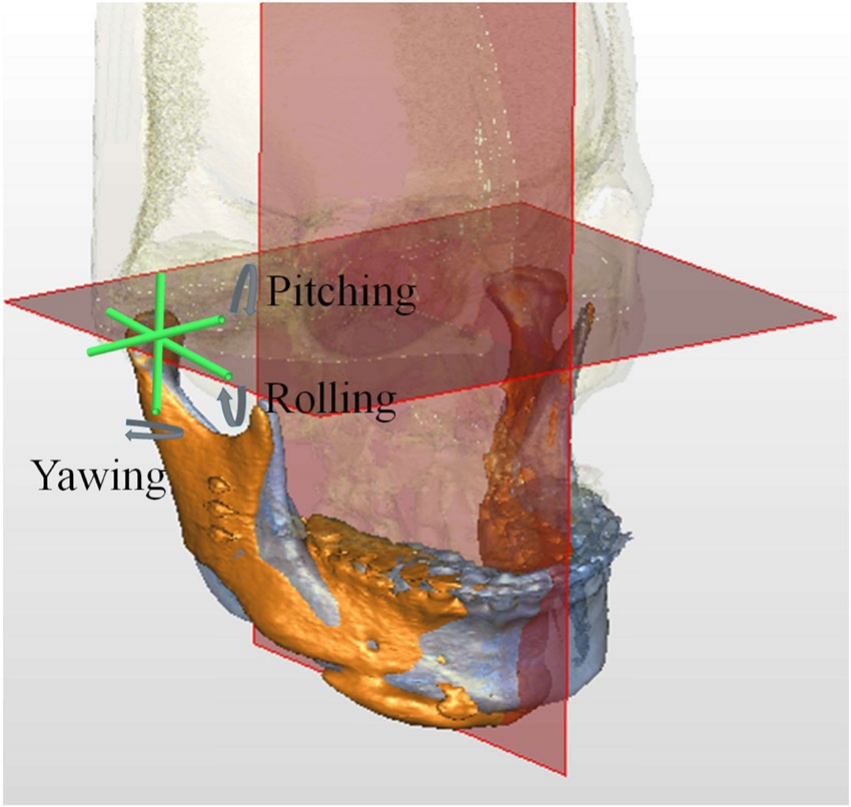
Rotations of right proximal segment in pitch (the anterior-posterior swing of the proximal segment), roll (the medial-lateral swing, or inward-outward movement of the proximal segment), and yaw (the left-right swing, or open-close movement of the proximal segment). Blue and orange colors represent T0 and T1 images, respectively.

**Figure 11:**
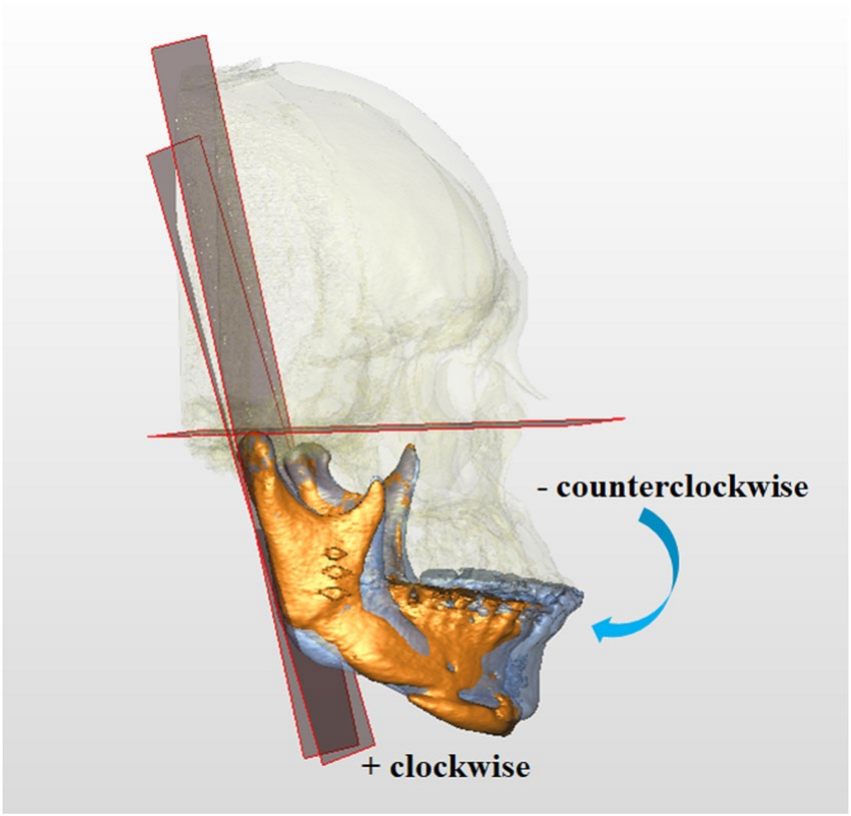
Rotations of right proximal segment in pitch direction. Negative values indicate counterclockwise rotation, whereas positive values clockwise rotation viewing from the axis (profile view). Blue and orange colors represent T0 and T1 images, respectively.

**Figure 12:**
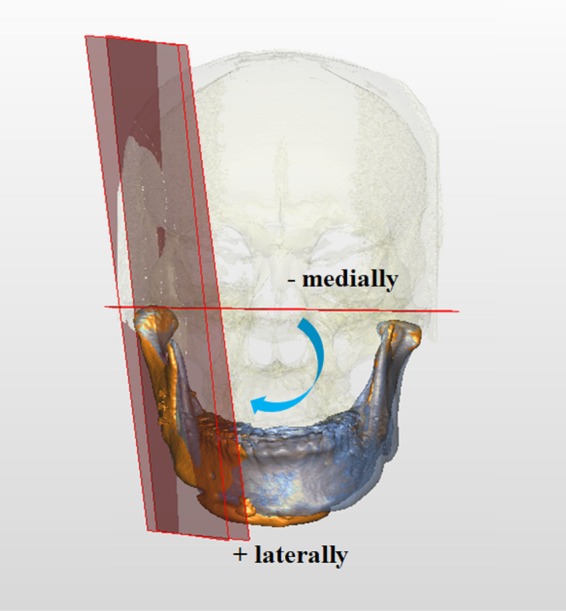
Rotations of right proximal segment in roll direction. Negative values indicate medial rotation, whereas positive values lateral rotation viewing from the axis (frontal view). Blue and orange colors represent T0 and T1 images, respectively.

**Figure 13:**
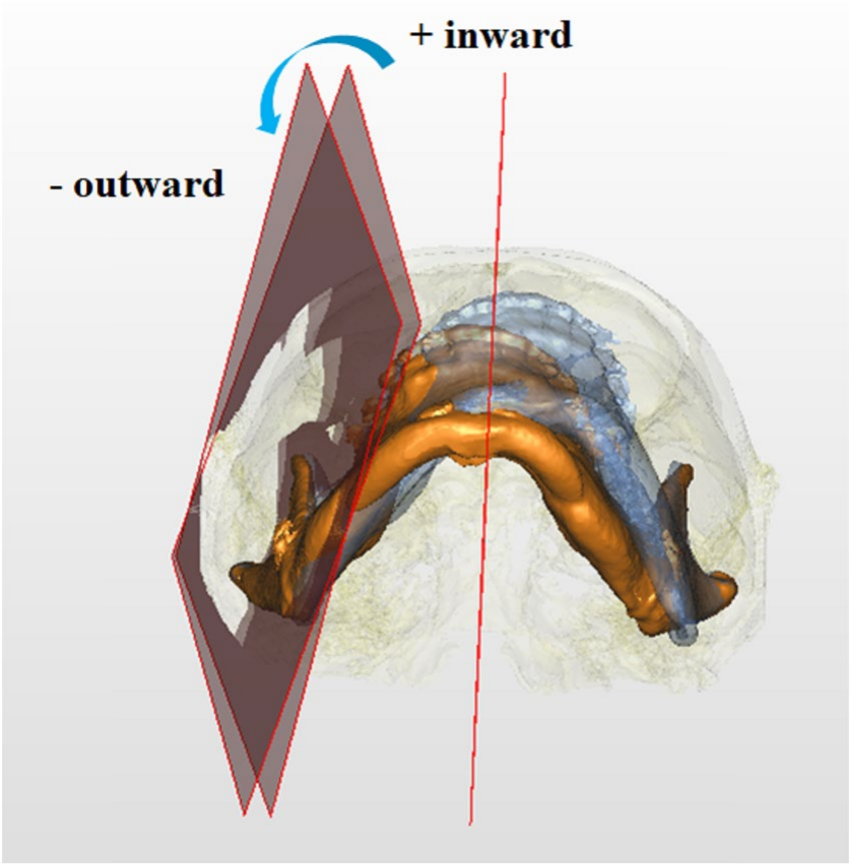
Rotations of right proximal segment in yaw direction. Negative values indicate outward rotation, whereas positive values inward rotation viewing from the axis (basal view). Blue and orange colors represent T0 and T1 images, respectively.

**Figure 14:**
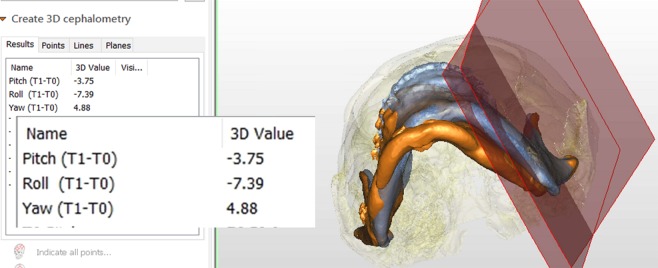
Superimposition and measurement of the rotation between the planes of proximal segment (PSPs) in T0 and T1 images (blue and orange colors, respectively).

To compare the positional differences of the proximal ramus segment between T0 and T1, image registration and superimposition of the forehead and orbital areas (non-operated parts) were performed using the best-fit method (Figs 6–8). Accuracy of the registration was verified by checking the distance color map (Geomagic 3D software, 3D Systems, Rock Hill, SC, USA) and RMSD (3dMD Vultus software, 3dMD LLC, Atlanta, USA) between the T0 and T1 images, with green color (Fig. 7) and value ≤0.5 mm (Fig. 8) considered acceptable to ensure that the corresponding reference areas had maximum precision^31,36^. After the registration, the FHP and midsagittal plane (MSP, Table 5) were employed on the skull model for comparisons (Fig. 9). To measure the change in the proximal ramus segment before and after skeletofacial surgery, the same coordinate system was applied for both T0 and T1 mandible models. The PSP in the T0 image was created and transferred to the corresponding position in the T1 image. Thereafter, the two PSPs were used to measure the angular differences by using SimPlant O&O software (Figs 10–14). The PSP reference plane representing the proximal segment was examined for its postoperative rotation in pitch, roll and yaw directions (Table 5) in the right and left sides (n = 31) and overall proximal ramus segments (n = 62). Using ten randomly selected patients (20 mandible sides), the same examiner repeated all landmark localizations and measurements per axis (x, y, and z) in an interval of 2 weeks for error assessment.

### The postoperative course

The patients with no intermaxillary fixation were admitted in regular ward for 2 days following the surgery. A liquid diet was advised in the first week, followed by a soft diet in the second week. All included patients were clinically examined for skeletofacial surgery-related complaints and complications based on established surgical and orthodontic appointments (1, 3, and 6 months postsurgery, and at least 1 year after debonding). The patient-centered outcome of the presence of TMJ-related symptoms and signs was also actively screened, including TMJ clicking, tenderness, mouth opening limitation, numbness, muscle tenderness, and headache^49^. Need for revision surgery was defined as any revisionary bone and/or soft tissue procedure requested or required to improve occlusal, maxillary, mandibular, and/or chin morphology within the follow-up.

### Statistical analysis

In the descriptive analysis, the mean was used for metric variables, and percentages were given for categorical variables. The data distribution was verified through the Kolmogorov-Smirnov test, and the paired t-test was adopted for statistical comparisons. Intra-examiner reliability was analyzed with intraclass correlation coefficients (ICCs) based on absolute agreement definition. Two-sided values of p < 0.05 were considered statistically significant. All analyses were performed using SPSS Version 19.0 (IBM Corp., Armonk, NY, USA).

## Supplementary information

Video S1

Supplementary Fig. S1

Supplementary Fig. S2

Supplementary Fig. S3
