# Supplementary material for: The outcome of skeletofacial reconstruction with mandibular rotation for management of asymmetric skeletal class III deformity: A three-dimensional computer-assisted investigation
Source: Sci Rep. 2019 Sep 16;9:13337. doi: 10.1038/s41598-019-49946-9 (PMC6746777; doi:10.1038/s41598-019-49946-9)
Supplement: Supplementary file 2 — Supplementary Fig. S1 [file 41598_2019_49946_MOESM2_ESM.pdf]

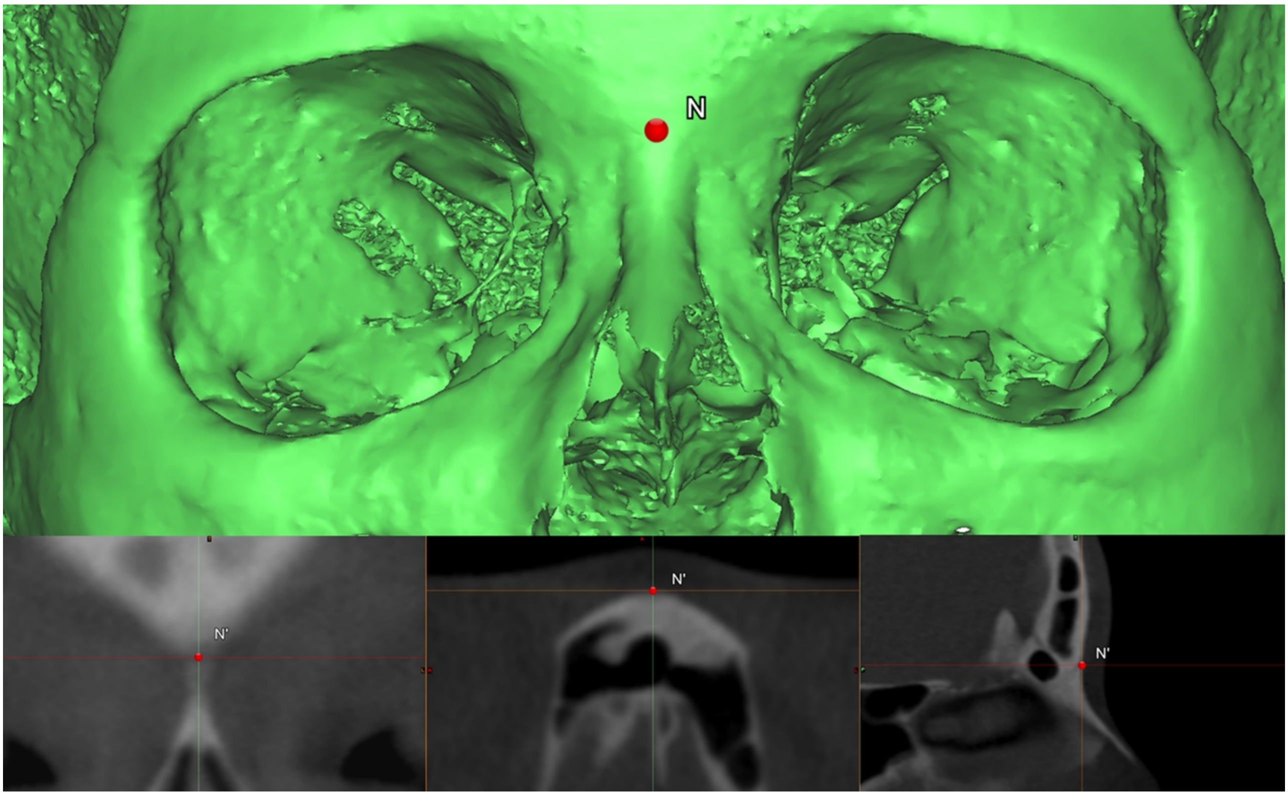

Supplementary Fig. S1. (*Top*) The nasal point (N) was identified on the CBCT-based 3D model, and (*bottom*) the nasal point (N') was identified in each slice.
