# Supplementary material for: The outcome of skeletofacial reconstruction with mandibular rotation for management of asymmetric skeletal class III deformity: A three-dimensional computer-assisted investigation
Source: Sci Rep. 2019 Sep 16;9:13337. doi: 10.1038/s41598-019-49946-9 (PMC6746777; doi:10.1038/s41598-019-49946-9)
Supplement: Supplementary file 3 — Supplementary Fig. S2 [file 41598_2019_49946_MOESM3_ESM.pdf]

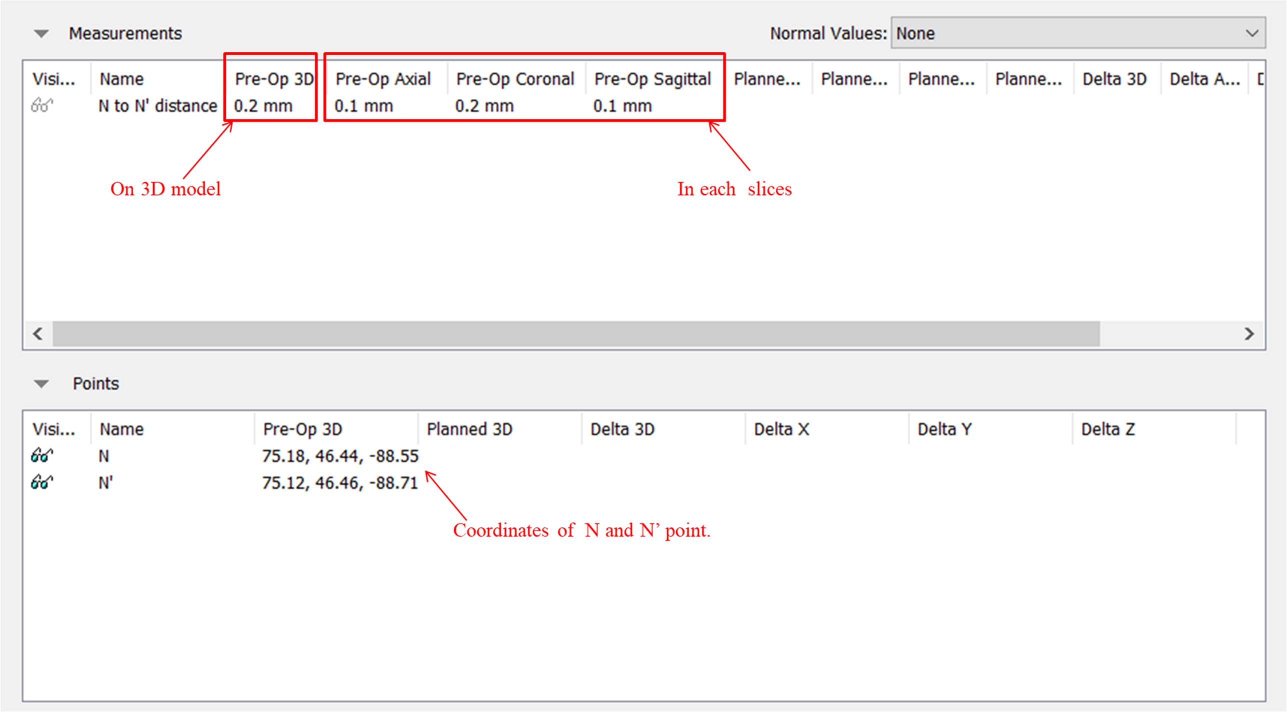

Supplementary Fig. S2. The distances between N and N' landmarks on the CBCT-based 3D model and in each slice were calculated (clinically acceptable value) to check the right threshold value for CBCT-based 3D model reconstruction.
