# Supplementary material for: The outcome of skeletofacial reconstruction with mandibular rotation for management of asymmetric skeletal class III deformity: A three-dimensional computer-assisted investigation
Source: Sci Rep. 2019 Sep 16;9:13337. doi: 10.1038/s41598-019-49946-9 (PMC6746777; doi:10.1038/s41598-019-49946-9)
Supplement: Supplementary file 4 — Supplementary Fig. S3 [file 41598_2019_49946_MOESM4_ESM.pdf]

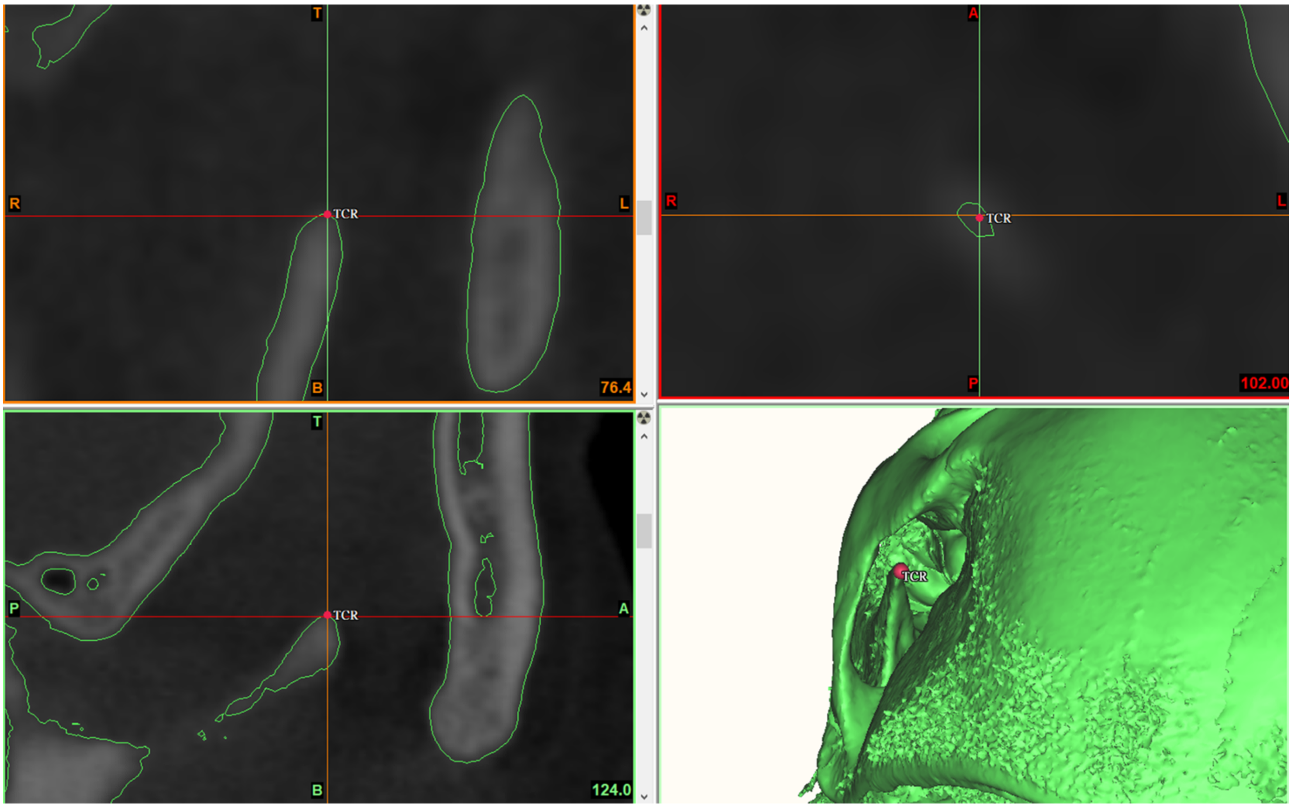

Supplementary Fig. S3. The surface of the CBCT-based 3D model with an interactive checking of the grayscale in each sliced image was performed to select and identify the TCR point. For TCR definition, please refer to Table 5.
